# Selecting interventions for a psychosocial support program for prostate cancer patients undergoing active surveillance: A modified Delphi study

**DOI:** 10.1002/pon.6053

**Published:** 2022-10-30

**Authors:** Kim Donachie, Marian Adriaansen, Minke Nieuwboer, Erik Cornel, Esther Bakker, Lilian Lechner

**Affiliations:** ^1^ Academy of Health HAN University of Applied Sciences Nijmegen The Netherlands; ^2^ Ziekenhuisgroep Twente Hengelo The Netherlands; ^3^ Faculty of Psychology and Educational Sciences Open University Heerlen The Netherlands

**Keywords:** active surveillance, cancer, diet, exercise, lifestyle, oncology, prostate cancer, psycho‐oncology, psychosocial support

## Abstract

**Objective:**

Curative treatment of low‐risk prostate cancer (LR‐PCa) does not improve cancer specific survival and active surveillance (AS) is recommended. Although AS is cost‐effective and reduces treatment‐related complications, it requires psychosocial support. Research on psychosocial interventions specifically focused on men undergoing AS is limited. Aim of this study is to reach consensus amongst relevant stakeholders on selecting interventions offering psychosocial support to PCa patients during AS.

**Methods:**

In accordance with the RAND/UCLA method, a modified Delphi approach was used to establish consensus on selecting interventions. During phase one, interventions were identified through a literature review and open survey among all participants. During phase two, three consensus rounds were conducted to rate potential interventions and obtain statistical consensus. The IQ healthcare consensus tool was used to calculate statistical consensus.

**Results:**

After the first consensus round, 31 participants scored individual interventions on relevance using a 9‐point Likert scale resulting in the selection of six interventions. During the second consensus round 13 discussion items were reviewed during a focus group. After the third consensus round, seven additional interventions were selected by 23 participants.

**Conclusions:**

In total, 13 interventions were selected for inclusion in a support program. This included four interventions within the domain *information and education*, three within *coping and* *support*, one intervention within *physical wellbeing* and four within the domain *lifestyle*.

## 1 BACKGROUND

Prostate cancer (PCa) incidence increases worldwide. Approximately 1.4 million men are diagnosed with PCa annually of which 375.000 men die.^1^ Due to aging and unhealthy lifestyle in the western population, a significant increase in PCa is expected within the next few decades.^2^ Low‐risk prostate cancer (LR‐PCa) entails 28% of all newly diagnosed PCa.^3^ Since curative treatment of LR‐PCa does not improve cancer specific survival, expectant management is safe and cost‐effective.^4^ It reduces treatment‐related complications such as erectile dysfunction, lower urinary tract symptoms (LUTS) and urinary incontinence.^5^, ^6^

Expectant management is divided into Watchful Waiting (WW) and Active Surveillance (AS). WW is a palliative option offered to men unable to undergo curative treatment due to comorbidity or limited life expectancy.^7^ AS is widely adopted as an acceptable management strategy in LR‐PCa and entails close monitoring with frequent medical examinations.^8^ Curative treatment is initiated upon detection of tumor progression.

During AS, frequent medical examinations and living with untreated disease can cause anxiety and uncertainty.^9^, ^10^ During the first year of AS, 30% of men risk developing anxiety.^11^ Some men are unable to cope with the psychological burden of AS. Approximately 5%–10% of men discontinue AS and pursue active treatment despite a lack of disease progression.^12^ Research indicates men undergoing AS need psychosocial support to promote psychological adaptability during AS.^9^, ^13^, ^14^ To prevent psychosocial problems, assessment of psychosocial support needs is important amongst all men undergoing AS and not merely those already experiencing problems. Psychosocial support in this study consists of all activities aimed at reducing the emotional burden associated with cancer by decreasing distress and improving effective coping strategies.

Within the psycho‐oncological research field, ample knowledge is available on the effects of psychosocial interventions on uncertainty, anxiety and distress in cancer patients. Based on findings from a scoping review, psychosocial support can decrease the psychosocial burden of AS and increase adherence.^15^ However, research on psychosocial interventions specifically focused on PCa patients undergoing AS is limited and mostly addresses stand‐alone interventions with a transitory effect.^15^, ^16^, ^17^

A comprehensive psychosocial support program, combining interventions with complementary characteristics is likely to lead to a broader and more sustainable effect. Such a program has the potential to optimize psychosocial support during AS and contribute to an effective and long‐term psychological adaptation, which results in decreased uncertainty, anxiety and increased AS adherence. Effects of such a multicomponent program have not been extensively researched. Components of such a program should be the results of a rigorous development process involving relevant stakeholders.

Therefore, aim of this study is to reach consensus amongst stakeholders on selecting interventions for a psychosocial support program offered to men with PCa undergoing AS.

## 2 METHODS

### 2.1 Design

A modified Delphi approach is used to establish consensus on interventions selected for inclusion in a support program. In contrast to the more exploratory character of classical Delphi methods, interventions in this study were previously identified through literature review and stakeholder consultation.^18^, ^19^

This study was conducted according to the RAND/UCLA Appropriateness Method.^20^ Ethical approval was obtained from the Open University Ethical Committee (ref.nr. U202104601).

The RAND/UCLA Appropriateness Method distinguishes two phases. During phase one, interventions are identified through a literature review and open survey among all participants. This survey identifies additional relevant interventions based on participants' experience and expertise, resulting in a complete and comprehensive list of potential interventions. During phase two, three consensus rounds are conducted to rate potential interventions and obtain statistical consensus.

### 2.2 Participants

A stakeholder analysis identified relevant stakeholders (e.g., patients, nurses, paramedics, physicians and mental health professionals). Purposive snowball sampling allowed inclusion of participants representing all stakeholders from various parts of the Netherlands. Potential participants were informed about the study and approached via e‐mail or telephone. Informed consent was obtained. At least five stakeholders per subgroup were enrolled to ensure stability of results.^21^

### 2.3 Phase 1: Selection of interventions

Between August and October 2020, a literature review was conducted.^15^ In addition to this review, relevant interventions described in a report from the Dutch Society of Psychosocial Oncology (NVPO) were added to the intervention list.^22^ All identified interventions were translated into Dutch and a list of potential interventions was presented to participants. Suggestions for additional interventions were discussed within the research team. Relevant interventions were added to the final intervention list. This final list was supplemented with a description, rationale, references and divided into four domains: *information and education, coping, physical wellbeing and lifestyle*.

### 2.4 Phase 2: Consensus rounds

During consensus round one, participants received the intervention list through LimeSurvey. Participants scored all interventions on relevance using a 9‐point Likert scale. *Relevance* in this study was defined as the extent to which participants found interventions essential for a support program. An open text field was added to each intervention and enabled participants to place remarks or suggestions. Participants were invited to complete the rating process between 9 July and 20 September 2021.

The second consensus round took place on 12 January 2022 and consisted of a 2 h focus group meeting. Due to COVID‐19 restrictions the meeting was organized online using Microsoft Teams. In accordance with the RAND/UCLA method the meeting was led by an independent experienced moderator (MN) and at least nine panel members were invited to participate.^20^ Using purposive sampling, a proportional representation of all stakeholders was ensured. Prior to the meeting, all focus group participants received a list of discussion items. After obtaining permission from participants the meeting was recorded. To emphasize the importance of a patient‐centered approach, each discussion item was introduced by the moderator after which patient perspectives were explicitly asked. Afterward, all other participants were asked to comment on the discussion item and more timid participants were actively invited to present their point of view. A researcher (KD) was present as an observant, taking field notes, writing down memo's and meeting minutes.

All study participants were briefed on the focus group results. During consensus round three, participants received a modified list of interventions through LimeSurvey. Again, all participants were invited to score interventions on relevance using a 9‐point Likert scale. An open text field was available for additional remarks. Participants were invited to complete the rating process between 3 March and 24 March 2022.

### 2.5 Data analysis

The IQ healthcare consensus tool was used to calculate statistical consensus after the first and third round.^23^ In accordance with the RAND/UCLA method, participants were invited to rate each intervention on relevance using a 9‐point Likert scale. Scores ranged from 1 *not relevant* to 9 *very relevant*. The RAND/UCLA method provides clear guidelines on execution of a consensus study and its procedures have been refined since its development in the 1980s resulting in a reliable and rigorous approach.^23^ The IQ healthcare consensus tool is based on the RAND/UCLA method and uses median and highest tertile scores.

After analyzing data from round one, a *selection*, *discussion* or *no selection* label was appointed to each intervention (see Table 1). A subgroup analysis was conducted to calculate consensus scores for the specific subgroups and identify between‐group differences. In addition, remarks and suggestions placed in open text fields were collected, and thematically analyzed. This provided insight into the rating process, existing concerns and motives. Results from the data‐analysis were discussed during a research team meeting. Interventions with a *discussion* or *no selection* label, substantial between‐group differences and important remarks and suggestions were selected by the research team as discussion item for the focus group meeting. Important results and findings from round one were presented to all study participants via e‐mail.

**TABLE 1:** Selected interventions first round

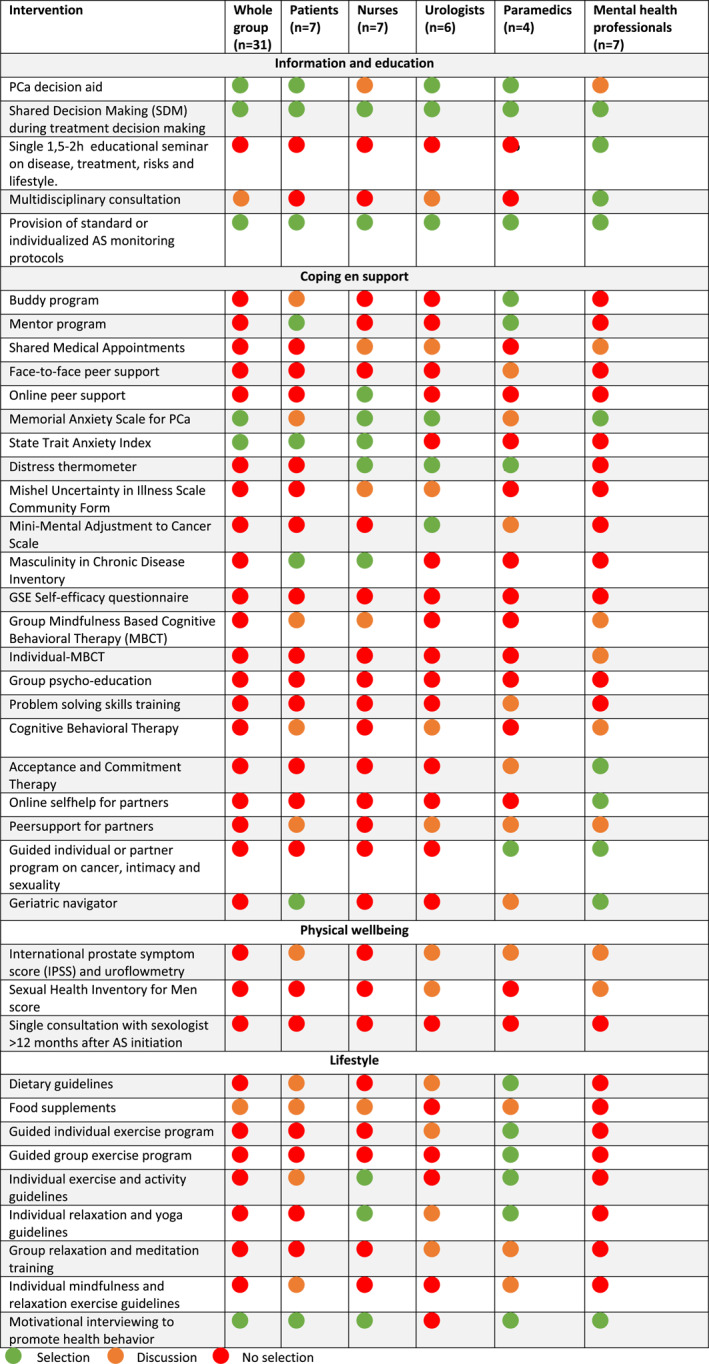

During the second round, discussion items were presented to panel members. Interventions were adjusted if focus group participants deemed it necessary or otherwise removed. The moderator checked for consensus on adjustment or removal. Consensus was established if >75% of participants agreed on adjustment or removal. During the focus group meeting, notes were collected by a researcher (KD) and discussed within the research team. Meeting notes and suggested adjustments were returned to all focus group participants for a member check. After approval, the list of interventions was modified in accordance with suggestions and findings from the first two rounds. After collection of data during the third consensus round, data were analyzed and a final consensus label was appointed to each intervention: *selection*, *discussion* or *no selection*.

### 2.6 Incomplete data handling

To prevent loss of valuable data, both complete and incomplete surveys were included in the analysis.

## 3 RESULTS

### 3.1 Selection of interventions

A total of 39 interventions were selected for the first consensus round (see Supporting Information S1). Interventions were divided into four domains (see Table 1). A literature review identified 13 interventions, 12 interventions were derived from the NVPO report and 14 resulted from the open survey amongst experts.

### 3.2 Participants

A total of 43 subjects were invited to participate in this study. This included 11 patients, nine nurses/nurse practitioners, 10 urologists, five paramedical and eight mental health professionals. Participation was declined by four subjects (one patient, two urologists and one nurse) due to practical reasons: time, personal circumstances. Approximately 80% (31/39) of subjects returned the survey. Three patients did not complete the entire survey. The primary reason provided was their self‐described inability to rate specific interventions.

### 3.3 First consensus round

After analyzing data from the first consensus round, statistical consensus on *selection*, *no selection* or *discussion* on all 39 interventions was established. Consensus on selection of six interventions was reached (see Table 1).

Subgroup analysis displayed important between‐group differences. Particularly in the domain *coping and support*, and *lifestyle*. For instance, various questionnaires were rated differently between subgroups. In addition, the importance of peersupport is valued conflictingly. Within the *lifestyle* domain there are large between and within‐group differences regarding relevance of exercise, relaxation and diet resulting in the labels *discussion* and *no selection* for the majority of interventions.

### 3.4 Thematic analysis of open text fields

Analysis of open text fields demonstrated three recurring themes: intervention feasibility, suggestions for adaption and tailored care. Feasibility concerns were often expressed in regard to sexological interventions. Especially costs and reimbursement possibilities were questioned. In addition, availability of certified sexologists was a major concern.

**Q1:** “*Costs! This is not reimbursed by health insurance companies and in a lot of hospitals sexologists are not present*.”

Time was also often addressed as a limiting factor. For instance in regard to multidisciplinary consultations.

**Q2:** “*Difficult, expensive and time‐consuming to get everyone together.*”

Often participants provided suggestions for adaption of interventions. For instance, provision of information through an educational seminar can be replaced by online information, complemented by individual consultations. With regard to facilitation of peersupport, it was suggested to combine peersupport with an activity to promote accessibility.

**Q3:** “ *…link this to something active, this makes talking easier.*”

Tailored or stepped care was also frequently recommended. Information and education should be adjusted to needs and characteristics of patients and involve partners. Emphasizing on understandable and just‐in‐time information. Interventions focusing on mindfulness, relaxation, and stress reduction should be provided to patients expressing unmet needs and should not be standard care.

**Q4:** “*Might be relevant for a subgroup of patients, good to have the possibility and offer it to particular men*.”

Although anonymous, analysis of open field texts also provided possible explanations for observed between‐group differences. Notable was a statement from a participant in relation to the use of mindfulness and stress‐reduction interventions.

**Q5:** “*Are you suitable for AS if you need support in coping with anxiety, uncertainty, or distress during AS? …. they should opt for active treatment*.”

The relationship between anxiety, uncertainty, and exercise was questioned by some participants. The importance of food and diet as a self‐management strategy to cope with the psychosocial burden of AS was recognized by some participants and doubted by others. Presence of sexological or urological problems in the AS population was not acknowledged by some participants. They linked these problems to patients in active treatment groups.

### 3.5 Second consensus round

The second consensus round consisted of an online focus group meeting. Out of the 12 invited focus group members, 11 participated. This included three patients, two nurse practitioners, two urologists, three paramedical caregivers and one mental health professional. One mental health professional was unable to attend. During the meeting, all 13 predetermined items, were discussed. Based on group consensus, four interventions were removed and nine interventions were adjusted (see Table 2).

**TABLE 2:** Discussion items focus group

Intervention	Discussion	Results
Information and education		
Single educational seminar	Feasibility, suggestions for adjustment, subgroup differences	Suggestions for adjustment
Multidisciplinary consultations	Suggestions for adjustment, subgroup differences	Suggestions for adjustment and separation into multidisciplinary medical and paramedical consultations
Coping and support		
Participation in buddy or mentor program	Relevance, desirability, subgroup differences	Removal
Shared medical appointments	Feasibility, suggestions for adjustment	Removal
Peer support for patients and/or partners	Suggestions for adjustment, subgroup differences	Suggestion for adjustment and separation into peer support online, face‐to‐face and involving partners
Questionnaires for anxiety, uncertainty, distress, masculinity, coping, self‐image, self‐efficacy	Feasibility, suggestions for adjustment, subgroup differences	Suggestion for adjustment
Mindfulness	Relevance, subgroup differences	Removal
Psycho‐education	Relevance, subgroup differences	Removal
Sexological screening and/or consultation	Feasibility, suggestions for adjustment	Suggestion for adjustment
Physical wellbeing		
Screening of lower urinary tract symptoms and erectile dysfunction	Feasibility, suggestions for adjustment	Suggestion for adjustment
Lifestyle		
Dietary recommendations and food supplements	Relevance, subgroup differences	Suggestion for adjustment and additional educational intervention
Exercise and physical activity guidelines	Suggestions for adjustment, subgroup differences	Suggestions for adjustment and additional supportive intervention
Relaxation, yoga, and meditation	Suggestions for adjustment	Suggestion for adjustment

### 3.6 Third consensus round

Based on results from the previous two rounds, an adapted list containing 14 interventions was returned to study participants (see Table 3). Out of the 39 participants, three declined participation due to practical reasons. The rating process was completed by 23/36 participants through LimeSurvey resulting in a 64% response. This included five patients, four nurse practitioners, five urologists, three paramedics, and five mental health professionals. After data‐analysis, statistical consensus on all 14 interventions was established. All 23 participants completed the survey. Consensus on additional selection of seven interventions was reached (see Table 3). After the third consensus round, seven remaining interventions were rejected based on their *discussion* or *no selection* label. Due to the small subgroup sizes a subgroup analysis was not performed.

**TABLE 3:** Selected interventions third round

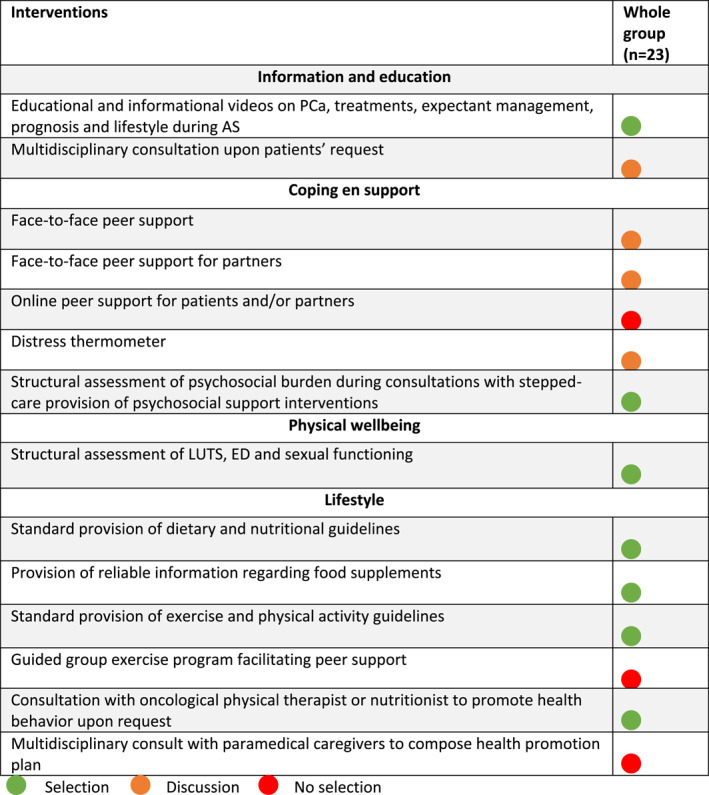

## 4 DISCUSSION

Aim of this study was to reach consensus amongst relevant stakeholders on selecting interventions for a psychosocial support program offered to PCa patients undergoing AS. Findings from this study suggest that this support program should include 13 interventions.

After round one, immediate consensus was reached on application of decision aids, SDM and provision of monitoring protocols. This demonstrates the widely shared desire to improve information and education during treatment decision making in LR‐PCa. The need for transparent, unambiguous, tailored information and education involving both patients, partners and family members has been underlined in previous literature.^9^, ^24^ This is becoming more important since AS is increasingly applied and also extends to patients with favorable intermediate risk PCa.^25^ However, merely implementing informational and educational interventions is not enough to actively involve and empower patients. Research suggests patient‐centered decision making also requires a behavior change in caregivers.^26^ A support program should therefore also include communication training for caregivers to break long‐standing interaction patterns.^27^

Both the Memorial Anxiety Scale for PCa and State Trait Anxiety Index were selected during the first consensus round. This illustrates the perceived importance of adequately detecting anxiety in men undergoing AS. An important trigger for anxiety and uncertainty during AS are recurring medical examinations.^28^ Presence thereof negatively impacts physical and psychosocial wellbeing.^29^ Standardized screening and adequate support upon detection need improvement.^10^, ^11^, ^12^, ^13^ Distress is often not expressed by PCa patients.^30^ Open communication and empathy, especially from male caregivers, helps men to openly address distress.^30^ During the second consensus round this topic was discussed extensively. A more structural and proactive approach in detecting mental or physical problems is required. Issues regarding masculinity, sexuality, intimacy and urinary function are not necessarily addressed by patients during routine consultations. Especially not, when initiative lies with the patient. This may be caused by stigmatization, which plays an important role in the lives of men with PCa.^31^ Adequate screening of physical and mental health needs and stepped individualized care were suggested during the focus group. This resulted in adjustment and selection of two interventions during the third consensus round: structural assessment of psychosocial and physical wellbeing (LUTS, ED, intimacy, and sexuality) during consultations. Upon detection of unmet needs, supportive interventions are provided by a caregiver or patients are referred to a specialist.

Additionally, prevention of mental and physical problems by supporting self‐management was perceived relevant. Although interventions directly aiming at lifestyle improvements were not selected during round one, supporting men expressing the need to improve health behavior was perceived important. Application of motivational interviewing to promote health behavior was selected during round one. Recently, there has been an increased interest in the relationship between PCa progression and health behavior during AS.^32^, ^33^, ^34^, ^35^ Research suggests men diagnosed with PCa frequently have a desire to adopt a healthier lifestyle.^36^ However, caregivers generally provide inadequate information and support. Healthy lifestyle modifications positively influence physical and mental wellbeing.^37^ It is suggested caregiver education is necessary to improve information delivery and overcome barriers, such as a perceived lack of evidence.^37^ Providing information regarding exercise and dietary guidelines, food supplements, relaxation techniques and health promotion was discussed during the focus group meeting. This resulted in adjustment and selection of the following interventions during consensus round three: standard provision of healthy dietary recommendations and guidelines, provision of reliable information regarding food supplements, standard provision of healthy exercise and physical activity recommendations and guidelines and consultation with oncological physical therapist or nutritionist to promote health behavior upon request.

### 4.1 Study limitations

Despite carefully designing this study, several limitations should be addressed. First, participants in this study were recruited using snowball sampling. Therefore, selection bias cannot be ruled out. Yet, a large and conscientious representation of all subgroups was purposively selected. This included participants from varying parts of the country and enrollment of health care professionals from larger and smaller institutions. The large response rates improved reliability of the results. However, a larger proportion of healthcare providers than patients were included. As a result, conclusions of this study may represent, to a greater extend, perspectives of healthcare providers.

Patients also addressed an important limitation by declaring they felt incapable of adequately scoring the relevance of certain interventions. This resulted in three incomplete surveys during round one. Although results from consensus round one may underrepresent patient perspectives, a conscious emphasis was placed on patient perspectives throughout this study. First, by including incomplete data from patients collected during the first round. Since consensus is calculated for each intervention separately, inclusion of incomplete surveys does not affect the calculated consensus scores. However, it does increase the risk of non‐response or participation bias. Second, emphasis on patient perspectives was ensured during the focus group meeting. A patient‐centered approach was used to actively engage and empower patients.

An additional important limitation was caused by COVID‐19 restrictions. Online focus groups impede live interaction between participants. However, the independent moderator focused on equal contribution of all participants and invited participants to speak freely, share thoughts and emotions expressed non‐verbally.

### 4.2 Clinical implications

Increased AS adoption, even within the intermediate risk population, is observed globally. AS is recommended as the preferred treatment option in low‐risk prostate cancer. The importance of AS, as part of the urological treatment palette, is growing. Resulting in a larger AS population. Establishing important preconditions for successful selection and adherence to AS is becoming progressively important. This emphasizes the need for a psychosocial support program in men undergoing AS. Assessment and identification of psychosocial problems and needs should be conducted amongst all men undergoing AS and not merely those already experiencing problems. Findings from this study suggest that such a support program should integrate the key elements comprising the 13 interventions as chosen. These interventions originate from research conducted in Germany, the United States of America, Canada, the United Kingdom, Australia and Japan, improving the generalizability of these findings.^15^ Future research should focus on the composition and operationalization of such a support program. Moreover, this study underlines the importance of a more patient‐centered approach to AS combining the implementation of patient‐orientated interventions as well as a change in caregiver attitudes and behaviors to promote open communication and patient empowerment.^38^

## 5 CONCLUSIONS

Based on this modified Delphi study, a total of 13 interventions were selected for inclusion in a support program for men with LR‐PCa undergoing AS. This included four interventions within the domain information and education, three interventions within the domain coping and support, one intervention within the domain physical wellbeing and four interventions within the domain lifestyle. Interventions originate from a literature review, the NVPO report and an open survey amongst experts.^15^, ^22^

## AUTHOR CONTRIBUTIONS

All authors have made substantive contributions to the conception and design, data collection, analysis and interpretation of data, draught and revision of the article. Those who have made substantive contributions to the article have been named as authors.

## CONFLICT OF INTEREST

The authors have no conflict of interest to declare.

## ETHICS STATEMENT

The study was conducted in accordance with the Declaration of Helsinki and Good Clinical Practice Guidelines. Ethical approval was obtained from the Open University Ethical Committee (ref.nr. U202104601) and this study was conducted in accordance with the Dutch Medical Research with Human Subjects Law.

## PATIENT AND PUBLIC INVOLVEMENT

Patients were actively involved in the conduct of this study. During the focus group meeting the moderator had an active role in the empowering of patients and emphasizing the patient perspective.

## Supporting information

Supporting Information S1

## Data Availability

The data that support the findings of this study are available from the corresponding author upon reasonable request.
