# Supplementary material for: Selecting interventions for a psychosocial support program for prostate cancer patients undergoing active surveillance: A modified Delphi study
Source: Psychooncology. 2022 Oct 30;31(12):2132–40. doi: 10.1002/pon.6053 (PMC10092864; doi:10.1002/pon.6053)
Supplement: Supplementary file 1 — Supporting Information S1 [file PON-31-2132-s001.docx]

**Supplemental material 1. Consensus labels IQ healthcare consensus tool**

|  | *>70% in the highest tertile* | *>30% in the lowest tertile and >30% in te highest tertile* | *<70% in the highest tertile* |
| --- | --- | --- | --- |
| *Median < 3* | *Discussion* | *Discussion* | *No selection* |
| *Median 4 < 7* | *Discussion* | *Discussion* | *No selection* |
| *Median > 8* | *Selection* | *Discussion* | *Discussion* |

**Supplemental material 2. Selected interventions overview box**

| Information and education   - (online) educational videos about prostate cancer, various treatment options, monitoring protocols, mortality, morbidity during AS, lifestyle advice - use of a prostate cancer decision aid - treatment decision in accordance with shared decision making (SDM) principles - provision of standard or individualised AS monitoring protocols   Coping and support   - application of Memorial Anxiety Scale for Prostate Cancer - application of State Trait Anxiety Index - structural assessment of psychosocial burden during consultations with stepped-care provision of psychosocial support interventions   Physical wellbeing   - structural assessment of LUTS, ED, problems with intimacy and sexuality during consultations   Lifestyle   - use of motivational interviewing to promote health behaviour - standard provision of healthy dietary recommendations and guidelines - provision of reliable information regarding food supplements - standard provision of healthy exercise and physical activity recommendations and guidelines - consultation with oncological physical therapist or nutritionist to promote health behaviour upon request |
| --- |

**Supplemental material 3. Complete intervention list with supporting evidence base**

General information

This research focusses on the development of a psychosocial support program for men with prostate cancer undergoing active surveillance. The purpose of this support program is to decrease the experienced psychosocial burden caused by active surveillance.

**Active surveillance**: a delayed treatment recommended for very low-risk prostate tumors. During active surveillance tumor progression is closely monitored using medical examinations such as: blood PSA-value, MRI-scan and prostate biopsies. (Kanker.nl, 2019)

This support program is developed to promote the adoption of effective coping strategies in men experiencing anxiety distress and uncertainty during active surveillance. Relevant (psychosocial) interventions are included in this support program. The program aims to promote self-management and disease control and to reduce uncertainty, anxiety and distress. The selected interventions contribute to:

- Emotional processing of the disease
- Coping with negative emotions caused by frequent medical examinations
- Support during treatment decision making
- Fear of cancer progression
- Psychological adaptation and coping with the disease and treatment

Interventions may also focus on problems in relationship with partner of family members, fatigue or the social impact of prostate cancer for instance related to work. (NVPO, 2015)

**Interventions:** non-pharmacological actions/applications that utilize psychological techniques such as education, training of coping skills, psychotherapy and/or relaxation therapy. These interventions are performed by a professional. Direct, face-to-face and interpersonal. The goal is to reduce emotional stress and improve quality of life in patients with cancer. (Faller, et al 2013)

In addition, this study also includes indirect and non-psychosocial interventions such as unsupervised self-management interventions, exercise, diet and/or complementary medicine as well as informational and educational strategies: i.e. brochures and videos.

During this consensus study experts are asked to determine the relevance of the different interventions for a psychosocial support program in men with prostate cancer during active surveillance.

**Relevance:** the extent to which the expert considers the intervention important or essential as part of a support program to reduce the psychosocial burden in men undergoing active surveillance. Do you consider the intervention valuable within a support program and does the use of the intervention as part of a program contribute to reducing anxiety, uncertainty and stress?

The aim of this questionnaire is to reach consensus amongst all participants of the expert group regarding the relevance of interventions as part of a support program for men with prostate cancer undergoing active surveillance. The results of this questionnaire form the input for a planned consensus meeting. The interventions are based on scientific literature and complemented by suggestions of participating experts. The interventions are divided into the following domains: information and education, coping and support, physical well-being, lifestyle.

Name

|  |
| --- |

Gender

|  |
| --- |

Organization

|  |
| --- |

Job description

|  |
| --- |

Domain 1: Information and education

# Intervention:

Standard application of a prostate cancer decision aid (brochure or website) after prostate cancer diagnosis. This provides the patient and family with information regarding individual risk, treatment options and (dis)advantages of various treatment options. A decision aid is used to guide the patient during treatment considerations and in making a treatment decision.

# Rationale:

There are various treatment options for patients with localized prostate cancer: active surveillance, surgery and radiotherapy.

Effective decision aids have been developed to support patients and care providers in making a treatment decision. However, its implementation in daily practice is lagging is not optimal.

A large proportion of care recipients are happy to participate in the decision-making process. A decision aid has a positive effect on the knowledge and satisfaction of patients.

van Tol-Geerdink, J. J., van Oort, I. M., Somford, D. M., Wijburg, C. J., Geboers, A., van Uden-Kraan, C. F., ... & Stalmeier, P. F. (2020). Implementation of a decision aid for localized prostate cancer in routine care: A successful implementation strategy. Health informatics journal, 26(2), 1194-1207.

Violette, P. D., Agoritsas, T., Alexander, P., Riikonen, J., Santti, H., Agarwal, A., ... & Tikkinen, K. A. (2015). Decision aids for localized prostate cancer treatment choice: systematic review and meta‐analysis. CA: a cancer journal for clinicians, 65(3), 239-251.

Do you consider the standard use of a prostate cancer decision aid relevant as an intervention to prevent or reduce anxiety, uncertainty and (dis)stress in patients with prostate cancer who are eligible for active surveillance?

|  |
| --- |

Remarks?

|  |
| --- |

Domain 1: Information and education

# Intervention:

Standard scheduling of a shared-decision making (SDM) consult with a nurse/nursing specialist in which SDM is used as an interview technique by the nurse to support the patient in making a treatment decision.

# Rationale:

The use of shared decision-making (SDM) as a methodical discussion technique is recommended for preference-sensitive medical decision moments. It promotes adherence and satisfaction. Patients with localized prostate cancer can choose between various treatment options: active surveillance, surgery and radiotherapy.

SDM is characterized by an open exchange of information and equality in the interaction between care recipient and care provider. The 7-step model and the 3 good questions are discussion tools to promote SDM. Decision aids and audio recording of conversations can be tools to facilitate SDM.

Martínez-González, N. A., Plate, A., Senn, O., Markun, S., Rosemann, T., & Neuner-Jehle, S. (2018). Shared decision-making for prostate cancer screening and treatment: a systematic review of randomised controlled trials. Swiss medical weekly, 148, w14584.

Would you consider the standard use of an SDM consult by a nurse relevant as an intervention to prevent or reduce anxiety, uncertainty and (dis)stress in patients with prostate cancer who are eligible for active surveillance?

|  |
| --- |

Remarks?

|  |
| --- |

Domain 1: Information and education

# Intervention:

A single educational seminar of 1.5-2 hours on prostate cancer, the various treatment options, the active surveillance policy, mortality and morbidity with active surveillance and information about lifestyle.

The seminar is offered periodically and can be attended by patients immediately after diagnosis or during active surveillance.

# Rationale:

Care recipients with prostate cancer need transparent, unambiguous information about active surveillance policy, the risks, advantages and disadvantages. The use of a single educational seminar for care recipients and their relatives leads to improved adherence and satisfaction among care recipients during active surveillance.

Kinsella, N., Beckmann, K., Cahill, D., Elhage, O., Popert, R., Cathcart, P., ... & Van Hemelrijck, M. (2019). A single educational seminar increases confidence and decreases dropout from active surveillance by 5 years after diagnosis of prostate cancer. European urology oncology, 2(4), 464-470.

Would you consider the use of a single educational seminar relevant as an intervention to prevent or reduce anxiety, uncertainty and (dis)stress in patients with prostate cancer who are eligible for active surveillance?

|  |
| --- |

Remarks?

|  |
| --- |

Domain 1: Information and education

# Intervention:

Offering a standard multidisciplinary consultation with the urologist, radiotherapist, (possibly psychologist) and oncology nurse/nurse specialist to discuss the individual medical situation and personal considerations. This consultation is offered in the decision-making phase after prostate cancer diagnosis. If there is a need, this consultation will be offered again <12 months after the start of active surveillance.

# Rationale:

Multidisciplinary consultations with a urologist, radiotherapist, oncology nurse and possibly a psychologist offer care recipients a more balanced perspective on the advantages and disadvantages of different treatment options. This helps care recipients to make a more informed decision that is in line with the care recipient's goals, wishes and preferences.

Kinsella, N., Beckmann, K., Cahill, D., Elhage, O., Popert, R., Cathcart, P., ... & Van Hemelrijck, M. (2019). A single educational seminar increases confidence and decreases dropout from active surveillance by 5 years after diagnosis of prostate cancer. European urology oncology, 2(4), 464-470.

Aizer, A. A., Paly, J. J., Zietman, A. L., Nguyen, P. L., Beard, C. J., Rao, S. K., ... & Efstathiou, J. A. (2012). Multidisciplinary care and pursuit of active surveillance in low-risk prostate cancer. Journal of Clinical Oncology, 30(25), 3071-3076.

Would you consider the standard provision of multidisciplinary consultations to be relevant as an intervention to prevent or reduce anxiety, uncertainty and (dis)stress in patients with prostate cancer who are eligible for active surveillance?

|  |
| --- |

Remarks?

|  |
| --- |

Domain 1: Information and education

# Intervention:

Make standardized/individualized AS monitoring protocols available and provide standardized monitoring protocols to patients with prostate cancer during active surveillance. This protocol makes it clear to the care recipient which examinations will take place at what time.

# Rationale:

Clear, reliable and transparent information is an important need of patients during active surveillance. Currently, patients sometimes experience the information provided as unclear and insufficiently transparent. This causes uncertainty. Making the monitoring protocols available, including information about follow-up examinations and appointments, can meet this need, according to Loeb et al (2018).

Loeb S, Curnyn C, Fagerlin A, et al. Informational needs during active surveillance for prostate cancer: a qualitative study. Patient Educ Couns. 2018;100(9):241-247. N.PAG-N.PAG

McIntosh, M., Opozda, M. J., Evans, H., Finlay, A., Galvão, D. A., Chambers, S. K., & Short, C. E. (2019). A systematic review of the unmet supportive care needs of men on active surveillance for prostate cancer. Psycho‐oncology, 28(12), 2307-2322.

Would you consider the provision of AS monitoring protocols relevant as an intervention to prevent or reduce anxiety, uncertainty and (dis)stress in patients with prostate cancer during active surveillance?

|  |
| --- |

Remarks?

|  |
| --- |

Domain 2: Coping and support

# Intervention:

Participation of care recipients during active surveillance in a buddy program. A buddy program is a form of peer contact in which two care recipients are linked together during active surveillance. They can offer each other practical and emotional support, exchange experiences and information.

# Rationale:

A buddy is a form of peer contact. The buddy is a reliable and credible peer. As an experience expert, he knows what active surveillance means for patient. A buddy often has more time for support than formal caregivers. The language used by a buddy often corresponds better than the language used by formal caregivers.

Research by Weber et al (2004, 2007) shows that participation in a buddy program can reduce feelings of depression and improve quality of life.

Weber, B. A., B. L. Roberts, M. Resnick, G. Deimling, J. A. Zauszniewski, C. Musil, et al. 2004. The effect of dyadic intervention on self-efficacy, social support, and depression for men with prostate cancer. Psychooncology 13:47–60.

Weber, B. A., B. L. Roberts, H. Yarandi, T. L. Mills, N. R. Chumbler, and C. Algood. 2007. Dyadic support and quality-of-life after radical prostatectomy. J. Mens Health Gend. 4:156–164

Would you consider participation in a buddy program relevant as an intervention to prevent or reduce anxiety, uncertainty and (dis)stress in patients with prostate cancer during active surveillance?

|  |
| --- |

Remarks?

|  |
| --- |

Domain 2: Coping and support

# Intervention:

Participation in a mentoring program. The mentor program is similar to a buddy program. However, this concerns an experience expert with more and/or long-term experience with active surveillance.

# Rationale:

A mentor program has a positive impact on mental resilience, quality of life and experienced distress in care recipients.

Geiger, A. M., Mullen, E. S., Sloman, P. A., Edgerton, B. W., & Petitti, D. B. (2000). Evaluation of a breast cancer patient information and support program. Effective clinical practice: ECP, 3(4), 157-165.

Ye, Z. J., Qiu, H. Z., Liang, M. Z., Liu, M. L., Li, P. F., Chen, P., ... & Zhao, J. J. (2017). Effect of a mentor-based, supportive-expressive program, Be Resilient to Breast Cancer, on survival in metastatic breast cancer: a randomised, controlled intervention trial. British journal of cancer, 117(10), 1486-1494.

Would you consider participation in a mentoring program relevant as an intervention to prevent or reduce anxiety, uncertainty and (dis)stress in patients with prostate cancer during active surveillance?

|  |
| --- |

Remarks?

|  |
| --- |

Domain 2: Coping and Support

# Intervention:

Shared Medical Appointments (SMAs) are follow-up visits in which a group of 8-12 patients come together for a check-up with the medical specialist during AS. In addition to a short individual consultation, a meeting of approximately 90 minutes will take place supervised by the urologist and nurse specialist/specialised nurse. In addition to the exchange of experiences, there is room for themes such as lifestyle, sexuality and health. For this, experts can be invited to attend the meeting.

# Rationale:

Shared Medical Appointments (SMAs)can contribute to an improved quality of life and increased satisfaction among care recipients. Within oncology, this form of consultation can lead to more integrative oncological care. In addition, SMAs can facilitate contact with peers in a low-threshold manner.

Thompson-Lastad, A., Atreya, C. E., Chao, M. T., Pollak, C., Dhruva, A., Santana, T., & Abrams, D. I. (2019). Improving access to integrative oncology through group medical visits: A pilot implementation project. The Journal of Alternative and Complementary Medicine, 25(7), 733-739.

Lavoie, J. G., Wong, S. T., Chongo, M., Browne, A. J., MacLeod, M. L., & Ulrich, C. (2013). Group medical visits can deliver on patient-centred care objectives: results from a qualitative study. BMC health services research, 13(1), 1-10.

Would you consider the use of SMAs relevant as an intervention to prevent or reduce anxiety, uncertainty and (dis)stress in patients with prostate cancer during active surveillance?

|  |
| --- |

Remarks?

|  |
| --- |

Domain 2: Coping and support

# Intervention:

Face-to-face discussion group for patients with prostate cancer during active surveillance. The group is intended to exchange experiences with others (recognition and recognition of experiences, feelings).

Themes that can be discussed are: self-image, body perception, fear of progression, relationship with partner, children and environment, sexuality, communication (own environment but also with doctors/therapists), resumption of work and future prospects.

# Rationale:

Contact with peers has a positive impact on quality of life and care recipient satisfaction. Face-to-face contact with peers seems particularly suitable for older and/or less educated care recipients.

Huber, J., Muck, T., Maatz, P., Keck, B., Enders, P., Maatouk, I., & Ihrig, A. (2018). Face-to-face vs. online peer support groups for prostate cancer: a cross-sectional comparison study. Journal of Cancer Survivorship, 12(1), 1-9.

Kronenwetter, C., Weidner, G., Pettengill, E., Marlin, R., Crutchfield, L., McCormac, P., ... & Ornish, D. (2005). A qualitative analysis of interviews of men with early stage prostate cancer: the Prostate Cancer Lifestyle Trial. Cancer nursing, 28(2), 99-107.

Would you consider the use of face-to-face discussion groups relevant as an intervention to prevent or reduce anxiety, uncertainty and (dis)stress in patients with prostate cancer during active surveillance?

|  |
| --- |

Remarks?

|  |
| --- |

Domain 2: Coping and support

# Intervention:

Online discussion groups. The group is intended for people who notice that they need to pay attention to what they have experienced or are experiencing and want to exchange experiences with others (recognition and recognition of experiences, feelings). Themes that can be discussed are: self-image, body perception, fear of progression, relationship with partner, children and environment, sexuality, communication (own environment but also with doctors/therapists), resumption of work and future prospects.

# Rationale:

Contact with peers has a positive impact on quality of life and care recipient satisfaction. Online contact with peers appears to be more suitable for younger care recipients and/or those with a higher education.

Huber, J., Muck, T., Maatz, P., Keck, B., Enders, P., Maatouk, I., & Ihrig, A. (2018). Face-to-face vs. online peer support groups for prostate cancer: a cross-sectional comparison study. Journal of Cancer Survivorship, 12(1), 1-9.

Would you consider the use of online peer contact relevant as an intervention to prevent or reduce fear, uncertainty and (dis)stress in patients with prostate cancer during active surveillance?

|  |
| --- |

Remarks?

|  |
| --- |

Domain 2: Coping and support

# Intervention:

Application of the Memorial Anxiety Scale for Prostate Cancer (MAX–PC). The MAX-PC is a measuring instrument developed for determining prostate cancer specific anxiety. The instrument may be better able to detect fluctuations in anxiety, for example around medical examinations and results (PSA, biopsy, MRI), than generic measures of anxiety.

The MAX-PC contains 18 items that are filled in by the care recipient. The questionnaire focuses on three components: general anxiety related to prostate cancer and/or treatment, PSA related anxiety and fear of progression.

# Rationale:

An anxiety assessment tool can be used to determine which men may need professional psychological support. This can reduce the perceived psychosocial burden. This can have a positive effect on quality of life and adherence to treatment.

van den Bergh, R. C., Korfage, I. J., Borsboom, G. J., Steyerberg, E. W., & Essink-Bot, M. L. (2009). Prostate cancer-specific anxiety in Dutch patients on active surveillance: validation of the memorial anxiety scale for prostate cancer. Quality of Life Research, 18(8), 1061-1066.

Roth, A., Nelson, C. J., Rosenfeld, B., Warshowski, A., O'shea, N., Scher, H., ... & Breitbart, W. (2006). Assessing anxiety in men with prostate cancer: further data on the reliability and validity of the Memorial Anxiety Scale for Prostate Cancer (MAX–PC). Psychosomatics, 47(4), 340-347.

Would you consider the use of the MAX-PC questionnaire relevant as an intervention to assess anxiety in patients with prostate cancer during active surveillance?

|  |
| --- |

Remarks?

|  |
| --- |

Domain 2: Coping and support

# Intervention:

Application of the State Trait Anxiety Index (STAI-6). The STAI-6 is a measurement tool for determining generic anxiety. The measuring instrument consists of six items with four answer options. A score between 1-4 can be awarded. The total score is between 20 and 80, with 80 representing maximum anxiety. A STAI-6 score of ≥44 classifies an individual as being highly anxious.

# Rationale:

An anxiety assessment tool can be used to determine which men may need professional psychological support. This can reduce the perceived psychosocial burden. This can have a positive effect on quality of life and adherence to treatment.

Venderbos, L. D., Van Den Bergh, R. C., Roobol, M. J., Schröder, F. H., Essink‐Bot, M. L., Bangma, C. H., & Korfage, I. J. (2015). A longitudinal study on the impact of active surveillance for prostate cancer on anxiety and distress levels. Psycho‐Oncology, 24(3), 348-354.

Would you consider the use of the STAI-6 questionnaire relevant as an intervention to assess anxiety in patients with prostate cancer during active surveillance?

|  |
| --- |

Remarks?

|  |
| --- |

Domain 2: Coping and support

# Intervention:

Applications of the distress thermometer. The Distress thermometer is a single item measurement tool that can be used to determine distress.

# Rationale:

A distress assessment tool can be used to determine which men may need professional psychological support. This can reduce the perceived psychosocial burden. This can have a positive effect on quality of life and adherence to treatment.

Chambers, S. K., Zajdlewicz, L., Youlden, D. R., Holland, J. C., & Dunn, J. (2014). The validity of the distress thermometer in prostate cancer populations. Psycho‐Oncology, 23(2), 195-203.

Would you consider the use of the Distress thermometer relevant as an intervention to determine (dis)stress in patients with prostate cancer during active surveillance?

|  |
| --- |

Remarks?

|  |
| --- |

Domain 2: Coping and support

# Intervention:

Application of the Mishel Uncertainty in Illness Scale Community Form (MUIS-C). The MUIS-C is a measuring instrument that can be used to determine uncertainty in men with prostate cancer. The MUIS-C consists of 23 items.

# Rationale:

An uncertainty assessment tool can be used to determine which men may need professional psychological support. This can reduce the perceived psychosocial burden. This can have a positive effect on quality of life and adherence to treatment.

Bailey Jr, D. E., Wallace, M., Latini, D. M., Hegarty, J., Carroll, P. R., Klein, E. A., & Albertsen, P. C. (2011). Measuring illness uncertainty in men undergoing active surveillance for prostate cancer. Applied Nursing Research, 24(4), 193-199.

Would you consider the use of the MUIS-C questionnaire relevant as an intervention to assess uncertainty in patients with prostate cancer during active surveillance?

|  |
| --- |

Remarks?

|  |
| --- |

Domain 2: Coping and support

# Intervention:

Application of the Mini-Mental Adjustment to Cancer scale (MINI-MAC). The MINI-MAC is a measuring instrument that can be used to determine coping strategies.

# Rationale:

With the help of a measuring instrument for determining coping strategies, it can be determined which men may need professional psychological support. This can reduce the perceived psychosocial burden. This can have a positive effect on quality of life and adherence to treatment.

Couper, J. W., Love, A. W., Duchesne, G. M., Bloch, S., Macvean, M., Dunai, J. V., ... & Kissane, D. W. (2010). Predictors of psychosocial distress 12 months after diagnosis with early and advanced prostate cancer. Medical Journal of Australia, 193, S58-S61.

Would you consider the use of the MINI-MAC questionnaire relevant as an intervention to determine coping strategies in patients with prostate cancer during active surveillance?

|  |
| --- |

Remarks?

|  |
| --- |

Domain 2: Coping and support

# Intervention:

Application of the Masculinity in Chronic Disease Inventory (MCD-I). The MCD-I has been specially developed for mapping the perceived masculinity of men with prostate cancer. This measuring instrument consists of 28 items and maps the following themes: physical strength and fitness, sexuality, emotional independence, optimism, willingness to take action.

# Rationale:

A measuring instrument for determining perceived masculinity can be used to determine which men may need professional psychological support with regard to masculinity and self-image. This can reduce the perceived psychosocial burden. This can have a positive effect on quality of life and adherence to treatment.

Bowie, J., Brunckhorst, O., Stewart, R., Dasgupta, P., & Ahmed, K. (2020). A systematic review of tools used to assess body image, masculinity and self‐esteem in men with prostate cancer. Psycho‐Oncology, 29(11), 1761-1771.

Would you consider the use of the MCD-I questionnaire relevant as an intervention to assess self-image and masculinity in patients with prostate cancer during active surveillance?

|  |
| --- |

Remarks?

|  |
| --- |

Domain 2: Coping and support

# Intervention:

Use of the Generalized Self-Efficacy Scale (GSE). The GSE is a measuring instrument to determine self-efficacy. The GSE contains 10 items.

# Rationale:

The GSE measures self-efficacy as the degree of perceived control, the capacity for self-management and the ability to use skills effectively under difficult circumstances. Self-monitoring and management is an important coping strategy for men with prostate cancer during active surveillance.

A self-efficacy measure can be used to determine which men may need professional psychological support. This can reduce the perceived psychosocial burden. This can have a positive effect on quality of life and adherence to treatment.

Curtis, R., Groarke, A., & Sullivan, F. (2014). Stress and self-efficacy predict psychological adjustment at diagnosis of prostate cancer. Scientific reports, 4(1), 1-5.

Would you consider the use of the GSE questionnaire relevant as an intervention to determine self-efficacy in patients with prostate cancer during active surveillance?

|  |
| --- |

Remarks?

|  |
| --- |

Domain 2: Coping and support

# Intervention:

Group Mindfulness Based Cognitive Behavioral Therapy (MBCT). The face-to-face MBCT consists of 8 weekly sessions of 2.5 hours and a silent day of 6 hours of meditation. Participants are also asked to practice 45 minutes at home six days a week and will receive CDs and exercises for this.

# Rationale:

The results from a recent meta-analysis (Ledesma & Kumano, 2009) and a systematic review (Shennan et al., 2011) examining mindfulness interventions in patients with cancer show that these interventions increase quality of life and reduce anxiety, depression and reduce stress-related complaints.

Nederlandse vereniging voor psycho-oncologische zorg. (2015). Interventions in de psychosociale zorg.

Would you consider the use of MBCT in a group relevant as an intervention to prevent or reduce anxiety, uncertainty and (dis)stress in patients with prostate cancer during active surveillance? .

|  |
| --- |

Remarks?

|  |
| --- |

Domain 2: Coping and support

# Intervention:

Individual MBCT is an adaptation of the MBCT group protocol. Like the mindfulness group training, I-MBCT consists of 8 weekly sessions, each of 60 minutes. During the sessions, breathing and body-oriented exercises are done and discussed, such as the body scan, sitting meditation, walking meditation and yoga. I-MBCT is not talk therapy.

# Rationale:

There are several studies and meta-analyses that show that group MBCT is effective in reducing psychological complaints in different (non-)clinical groups, including people with cancer - Carlson et al., 2013; Fjorback et al., 2011; Hoffman et al, 2010, Matchim et al, 2011). Own pilot research has shown that I-MBCT is effective in reducing psychological complaints such as depressive symptoms (Schroevers et al., 2013)

Nederlandse vereniging voor psycho-oncologische zorg. (2015). Interventions in de psychosociale zorg.

Would you consider the use of individual MBCT relevant as an intervention to prevent or reduce anxiety, uncertainty and (dis)stress in patients with prostate cancer during active surveillance?

|  |
| --- |

Remarks?

|  |
| --- |

Domain 2: Coping and support

# Intervention:

Psycho-education in groups. The program consists of nine meetings. The first four sessions are weekly, the next four sessions are biweekly. A final meeting is a return session two months after the eighth meeting. Sessions last 2.5 hours each.

Psycho-educational modules are alternated per session and include: breathing and relaxation, rational emotive techniques, social skills and assertiveness, visualization exercises and medical education.

Rationale:
Since 2005, the group program has been offered in a research context at the LUMC to women with breast cancer after curative treatment. Results showed that scores for emotional complaints (anxiety and depression) decreased significantly immediately after the course and that these scores remained the same in the following year. It also appeared that participants experienced fewer negative emotions with regard to their cancer. Quality of life scores, especially emotional, social and role functioning, improved during the course and remained stable thereafter.

Nederlandse vereniging voor psycho-oncologische zorg. (2015). Interventions in de psychosociale zorg.

Would you consider the use of psycho-education in a group to be relevant as an intervention to prevent or reduce fear, uncertainty and (dis)stress in care recipients with prostate cancer during active surveillance?

|  |
| --- |

Remarks?

|  |
| --- |

Domain 2: Coping and support

# Intervention:

Problem solving skills training. The idea behind the training is that the more effective patients are at solving or coping with stressful problems in daily life, the more likely they are to experience less stress. People can view problems in two different ways, namely with a positive or a negative problem orientation. In addition, three styles of problem solving can be defined: the rational, impulsive, or avoidant style. To promote successful problem solving, the aim is for a person to learn to apply problem-solving techniques rationally, systematically and skilfully.

# Rationale:

There are various studies and meta-analyses that show that problem solving therapy works for a diverse range of complaints and target groups, for an overview see Cuijpers et al. (2007) and Malouff et al. (2007). The research by Kinsella (2019) shows that prostate cancer patients who have a positive attitude during active surveillance experience less stress. The 'doing something extra' strategy in the study by Oliffe (2009) is also associated with more effective coping during active surveillance.

Nederlandse vereniging voor psycho-oncologische zorg. (2015). Interventions in de psychosociale zorg.

Would you consider the use of problem-solving skills training relevant as an intervention to prevent or reduce fear, uncertainty and (dis)stress in patients with prostate cancer during active surveillance?

|  |
| --- |

Remarks?

|  |
| --- |

Domain 2: Coping and support

# Intervention:

Cognitive behavioral therapy. The intervention concerns an individual cognitive behavioral intervention for anxiety, depression (adjustment disorders or chronic fatigue). The treatment (blended-care) consists of 8 contact moments with a psychologist, in which the focus is mainly on thoughts and behaviors associated with certain emotions. A number of these contacts will take place via the internet (‘e-consultation’) or by telephone. The other contacts are face to face. During the treatment, the factors that provoke or maintain fear will be examined.

# Rationale:

Cognitive behavioral therapy can be used with care recipients with anxiety complaints to teach them to deal with anxious thoughts and feelings.

Nederlandse vereniging voor psycho-oncologische zorg. (2015). Interventions in de psychosociale zorg.

Would you consider the use of cognitive behavioral therapy relevant as an intervention to prevent or reduce anxiety, uncertainty and (dis)stress in patients with prostate cancer during active surveillance?

|  |
| --- |

Remarks?

|  |
| --- |

Domain 2: Coping and support

# Intervention:

Acceptance and Commitment Therapy (ACT) is a form of behavioral therapy that focuses on accepting emotions and thoughts that prostate cancer and active surveillance can cause. In contrast to cognitive behavioral therapy, it does not focus on the content of thoughts, but on the relationship with those thoughts. ACT is about letting go of control and accepting your thoughts and feelings.

# Rationale:

Acceptance and Commitment therapy can be used with care recipients with, among other things, anxiety complaints to teach them to deal with negative emotions such as fear and distress. By accepting these emotions instead of avoiding or fighting them, space and energy is created for important things in life.

Fashler, S. R., Weinrib, A. Z., Azam, M. A., & Katz, J. (2018). The use of acceptance and commitment therapy in oncology settings: a narrative review. Psychological reports, 121(2), 229-252.

Would you consider the use of ACT relevant as an intervention to prevent or reduce fear, uncertainty and (dis)stress in patients with prostate cancer during active surveillance?

|  |
| --- |

Remarks?

|  |
| --- |

Domain 2: Coping and support

# Intervention:

Online self-help for partners of prostate cancer patients during active surveillance. Based on the course 'Hold on, to each other'. The course is a self-help course consisting of 6 basic lessons, each dealing with a theme: (1) Recognizing and dealing with emotions; (2) Strengthening resilience; (3) The influence of negative thoughts; (4) Recognizing values in life and in the relationship; (5) The importance of communication; (6) Be kind to oneself.

# Rationale:

Partners of patients with prostate cancer during active surveillance are an important source of support. In addition, a positive attitude of partners towards the active surveillance policy is an important condition for adherence to therapy. Research by Kinsella (2018) shows that partners often experience more tension and uncertainty about the treatment choice than the care recipients themselves. A course for partners of care recipients with prostate cancer during active surveillance can help them to (continue to) be a positive source of support for the care recipient.

Based on the 'Hold on, to each other' program. (Köhle, Drossaert, Schreurs, Hagedoorn, van Uden, Verdonck-de Leeuw & Bohlmeijer, in preparation; Köhle, Drossaert, Van Uden, Verdonck-de Leeuw & Bohlmeijer, in preparation).

Nederlandse vereniging voor psycho-oncologische zorg. (2015). Interventions in de psychosociale zorg.

Kinsella, N., Stattin, P., Cahill, D., Brown, C., Bill-Axelson, A., Bratt, O., ... & Van Hemelrijck, M. (2018). Factors influencing men's choice of and adherence to active surveillance for low-risk prostate cancer: a mixed-method systematic review. European urology, 74(3), 261-280.

Would you consider the use of an online self-help program for partners of patients with prostate cancer relevant as an intervention to prevent or reduce anxiety, uncertainty and (dis)stress in patients with prostate cancer during active surveillance?

|  |
| --- |

Remarks?

|  |
| --- |

Domain 2: Coping and support

# Intervention:

Peer support contact for partners of prostate cancer patients during active surveillance. Meeting group for partners of people with cancer with the aim of providing support to often heavily burdened partners through psycho-education and recognition and recognition through contact with fellow sufferers.

# Rationale:

Peer support is supportive. During the diagnosis and treatment choice phase, partners of prostate cancer patients experience more tension and uncertainty than the care recipient himself (Kinsella, 2018). In addition, the support of partners is an important condition for adherence to therapy during active surveillance.

Nederlandse vereniging voor psycho-oncologische zorg. (2015). Interventions in de psychosociale zorg.

Kinsella, N., Stattin, P., Cahill, D., Brown, C., Bill-Axelson, A., Bratt, O., ... & Van Hemelrijck, M. (2018). Factors influencing men's choice of and adherence to active surveillance for low-risk prostate cancer: a mixed-method systematic review. European urology, 74(3), 261-280.

Would you consider the use of peer contact for partners of care recipients with prostate cancer during active surveillance relevant as an intervention to prevent or reduce fear, uncertainty and (dis)stress in care recipients with prostate cancer during active surveillance?

|  |
| --- |

Remarks?

|  |
| --- |

Domain 2: Coping

# Intervention:

Cancer, intimacy and sexuality. This treatment is supervised by a sexologist. Ten different treatment modules are available within the program, of which approximately four or five modules are chosen for each care recipient that match the specific problems. The time investment per week is approximately 1.5 hours (30 minutes reading information texts, 30 minutes for assignments and 30 minutes for writing to the sexologist).

# Rationale:

Prostate cancer can affect perceived sexuality, masculinity and intimacy. For some care recipients it is difficult to discuss these problems in the doctor's office. Guidance by an independent specialist can make discussing questions and problems regarding sexuality, masculinity and intimacy more accessible.

Sadovsky R, Basson R, Krychman M et al. Cancer and sexual problems. J Sex Med. 2010;7:349-373.

Nederlandse vereniging voor psycho-oncologische zorg. (2015). Interventions in de psychosociale zorg.

Would you consider the use of a 'Cancer, intimacy and sexuality' program relevant as an intervention to prevent or reduce fear, uncertainty and (dis)stress in care recipients with prostate cancer during active surveillance?

|  |
| --- |

Remarks?

|  |
| --- |

Domain 3: Lichamelijk welzijn

# Intervention:

The Geriatric Navigator is an online screening tool for patients with prostate cancer aged 70 years and older. It is a specific geriatric case history. In addition to any urological problems, it helps the care provider to map the vulnerability and problem areas of older people with cancer in both psychosocial and somatic areas.

# Rationale:

Hamaker, M. E., Schiphorst, A. H., ten Bokkel Huinink, D., Schaar, C., & van Munster, B. C. (2014). The effect of a geriatric evaluation on treatment decisions for older cancer patients–a systematic review. Acta Oncologica, 53(3), 289-296.

Would you consider the use of the geriatric navigator in patients with prostate cancer of 70 years or older relevant as an Intervention to prevent or reduce anxiety, uncertainty and (dis)stress in patients with prostate cancer during active surveillance?

|  |
| --- |

Remarks?

|  |
| --- |

Domain 3: Physical well-being

# Intervention:

Periodic administration of the International Prostate Symptom Score (IPSS) questionnaire and the performance of uroflowmetry examinations for the evaluation of LUTS complaints.

# Rationale:

There is a correlation between the number of prostate biopsies, LUTS and erectile dysfunction. The presence of LUTS and erectile dysfunction has a negative effect on quality of life.

Fujita, K., Landis, P., McNeil, B. K., & Pavlovich, C. P. (2009). Serial prostate biopsies are associated with an increased risk of erectile dysfunction in men with prostate cancer on active surveillance. The Journal of urology, 182(6), 2664-2669.

Soloway, M. S., Soloway, C. T., Eldefrawy, A., Acosta, K., Kava, B., & Manoharan, M. (2010). Careful selection and close monitoring of low-risk prostate cancer patients on active surveillance minimizes the need for treatment. European urology, 58(6), 831-835.

Would you consider the periodic use of the IPSS questionnaire and the uroflowmetry study relevant as an intervention to prevent or reduce anxiety, uncertainty and (dis)stress in patients with prostate cancer during active surveillance?

|  |
| --- |

Remarks?

|  |
| --- |

Domain 3: Physical well-being

# Intervention:

Periodic use of the Sexual Health Inventory for Men Score and International Index Erectile Function (IIEF) to identify sexual problems and erectile dysfunction.

# Rationale:

There is a correlation between the number of prostate biopsies, LUTS and erectile dysfunction. The presence of LUTS and erectile dysfunction has a negative effect on quality of life

Fujita, K., Landis, P., McNeil, B. K., & Pavlovich, C. P. (2009). Serial prostate biopsies are associated with an increased risk of erectile dysfunction in men with prostate cancer on active surveillance. The Journal of urology, 182(6), 2664-2669.

Soloway, M. S., Soloway, C. T., Eldefrawy, A., Acosta, K., Kava, B., & Manoharan, M. (2010). Careful selection and close monitoring of low-risk prostate cancer patients on active surveillance minimizes the need for treatment. European urology, 58(6), 831-835.

Would you consider the use of the Sexual Health Inventory for Men Score and IIEF relevant as an intervention to prevent or reduce anxiety, uncertainty and (dis)stress in patients with prostate cancer during active surveillance?

|  |
| --- |

Remarks?

|  |
| --- |

Domain 3: Physical well-being

# Intervention:

Standard consultation with a sex therapist during the first 12 months of AS.

# Rationale:

Problems in the field of sexuality are not always discussed. Prostate cancer can influence the experienced sexuality and the development of sexual problems.

Palacios, L. A. G., Krouwel, E. M., den Oudsten, B. L., Den Ouden, M. E., Kloens, G. J., van Duijn, G., ... & Elzevier, H. W. (2018). Suitable sexual health care according to men with prostate cancer and their partners. Supportive Care in Cancer, 26(12), 4169-4176.

Do you consider the use of a standard consultation with the sexologist in the first 12 months after the start of active surveillance relevant as an intervention to prevent or reduce anxiety, uncertainty and (dis)stress in patients with prostate cancer during active surveillance?

|  |
| --- |

Remarks?

|  |
| --- |

Domain 4: Lifestyle

# Intervention:

Providing dietary guidelines that may reduce the risk of prostate cancer progression. This means: increased soy intake, increased chicken/poultry intake instead of red meat, increased vegetable intake, increased fish intake, increased green tea intake, increased red wine intake, increased linseed intake, increased pomegranate intake . Avoiding meat, fried foods, dairy products and carbohydrates.

# Rationale:

Limited evidence is available that certain dietary guidelines and nutritional supplements may reduce the risk of prostate cancer progression. In addition, diet and nutritional supplements provide an important self-management strategy for men with prostate cancer during active tracking. A 'doing something extra' coping strategy is associated with a more positive experience during active surveillance and a higher adherence to therapy.

Kenfield, S. A., Chang, S. T., & Chan, J. M. (2007). Diet and lifestyle interventions in active surveillance patients with favorable-risk prostate cancer. Current treatment options in oncology, 8(3), 173-196.

Berg, C. J., Habibian, D. J., Katz, A. E., Kosinski, K. E., Corcoran, A. T., & Fontes, A. S. (2016). Active holistic surveillance: the nutritional aspect of delayed intervention in prostate cancer. Journal of nutrition and metabolism, 2016.

Would you consider the use of dietary guidelines relevant as an intervention to prevent or reduce fear, uncertainty and (dis)stress in patients with prostate cancer during active surveillance?

|  |
| --- |

Remarks?

|  |
| --- |

Domain 4: Lifestyle

# Intervention:

Use of dietary supplements with a (possible) positive effect on the risk of prostate cancer progression. These include: broccoli extract, omega 3, zyflamed, vitamin D3, genikinoko, AHCC, lycocell, tumeric, pommegranate.

# Rationale:

Limited evidence is available that certain dietary guidelines and nutritional supplements may reduce the risk of prostate cancer progression. In addition, diet and nutritional supplements provide an important self-management strategy for men with prostate cancer during active tracking. A 'doing something extra' coping strategy is associated with a more positive experience during active surveillance and a higher adherence to therapy.

Kenfield, S. A., Chang, S. T., & Chan, J. M. (2007). Diet and lifestyle interventions in active surveillance patients with favorable-risk prostate cancer. Current treatment options in oncology, 8(3), 173-196.

Sumiyoshi, Y., Hashine, K., Kakehi, Y., Yoshimura, K., Satou, T., Kuruma, H., ... & Shinohara, N. (2010). Dietary administration of mushroom Mycelium extracts in patients with early stage prostate cancers managed expectantly: A phase II study. Japanese journal of clinical oncology, 40(10), 967-972.

Thomas, R., Williams, M., Sharma, H., Chaudry, A., & Bellamy, P. (2014). A double-blind, placebo-controlled randomised trial evaluating the effect of a polyphenol-rich whole food supplement on PSA progression in men with prostate cancer—the UK NCRN Pomi-T study. Prostate cancer and prostatic diseases, 17(2), 180-186.

Would you consider the use of nutritional supplements relevant as an intervention to prevent or reduce fear, uncertainty and (dis)stress in patients with prostate cancer during active surveillance?

|  |
| --- |

Remarks?

|  |
| --- |

Domain 4: Lifestyle

# Intervention:

Use of a guided individual exercise program. High-intensity interval training (HITT) 3 times a week for 28–40 min/session for 12 weeks.

# Rationale:

Limited evidence is available that active exercise and intense physical activity can reduce the risk of prostate cancer progression. Exercise has a direct positive effect on anxiety symptoms. In addition, sports and exercise provide an important self-management strategy for men with prostate cancer during active surveillance. A 'doing something extra' coping strategy is associated with a more positive experience during active surveillance and a higher adherence to therapy.

Galvão, D. A., Hayne, D., Frydenberg, M., Chambers, S. K., Taaffe, D. R., Spry, N., ... & Newton, R. U. (2018). Can exercise delay transition to active therapy in men with low-grade prostate cancer? A multicentre randomised controlled trial. BMJ open, 8(4).

McIntosh, M., Opozda, M., Galvão, D. A., Chambers, S. K., & Short, C. E. (2019). Identifying the exercise-based support needs and exercise programme preferences among men with prostate cancer during active surveillance: A qualitative study. European Journal of Oncology Nursing, 41, 135-142.

Kang, D. W., Fairey, A. S., Boulé, N. G., Field, C. J., & Courneya, K. S. (2019). Exercise duRing Active Surveillance for prostatE cancer—the ERASE trial: a study protocol of a phase II randomised controlled trial. BMJ open, 9(7), e026438.

Would you consider the use of a supervised individual exercise program relevant as an intervention to prevent or reduce fear, uncertainty and (dis)stress in patients with prostate cancer during active surveillance?

|  |
| --- |

Remarks?

|  |
| --- |

Domain 4: Lifestyle

# Intervention:

Use of a guided exercise program in groups. High-intensity interval training (HITT) 3 times a week for 28–40 min/session for 12 weeks.

# Rationale:

Limited evidence is available that active exercise and intense physical activity can reduce the risk of prostate cancer progression. Exercise has a direct positive effect on anxiety symptoms. In addition, sports and exercise provide an important self-management strategy for men with prostate cancer during active surveillance. A 'doing something extra' coping strategy is associated with a more positive experience during active surveillance and a higher adherence to therapy. Exercising in a group also facilitates contact with peers in an accessible way.

Galvão, D. A., Hayne, D., Frydenberg, M., Chambers, S. K., Taaffe, D. R., Spry, N., ... & Newton, R. U. (2018). Can exercise delay transition to active therapy in men with low-grade prostate cancer? A multicentre randomised controlled trial. BMJ open, 8(4).

Kang, D. W., Fairey, A. S., Boulé, N. G., Field, C. J., & Courneya, K. S. (2019). Exercise duRing Active Surveillance for prostatE cancer—the ERASE trial: a study protocol of a phase II randomised controlled trial. BMJ open, 9(7), e026438.

Would you consider the use of a supervised group exercise program to be relevant as an intervention to prevent or reduce anxiety, uncertainty and (dis)stress in patients with prostate cancer during active surveillance?

|  |
| --- |

Remarks?

|  |
| --- |

Domain 4: Lifestyle

# Intervention:

Providing exercise for unguided and individual exercise.

Advice per week 1000 MET. 1 MET is approximately 3.5 milliliters or oxygen consumption per kilogram of body weight. 150 minutes of medium-intensity aerobic activity is approximately equivalent to 500 MET.

# Rationale:

Limited evidence is available that active exercise and intense physical activity can reduce the risk of prostate cancer progression. Exercise has a direct positive effect on anxiety symptoms. In addition, sports and exercise provide an important self-management strategy for men with prostate cancer during active surveillance. A 'doing something extra' coping strategy is associated with a more positive experience during active surveillance and a higher adherence to therapy.

Papadopoulos, E., Alibhai, S. M., Doré, I., Matthew, A. G., Tomlinson, G. A., Nesbitt, M., ... & Santa Mina, D. (2020). Associations between self‐reported physical activity, quality of life, and emotional well‐being in men with prostate cancer on active surveillance. Psycho‐oncology, 29(6), 1044-1050.

Would you consider the use of exercise regulations relevant as an intervention to prevent or reduce anxiety, uncertainty and (dis)stress in patients with prostate cancer during active surveillance?

|  |
| --- |

Remarks?

|  |
| --- |

Domain 4: Lifestyle

# Intervention:

Providing relaxation and yoga exercises so that care recipients can apply them unsupervised, individually if necessary (in case of increased stress / tension) or as a fixed daily ritual.

# Rationale:

Relaxation and yoga exercises can help patients with prostate cancer to find physical and mental relaxation in daily life during active surveillance. The course offers tools on how to deal with worry and tension.

Oliffe, J. L., Davison, B. J., Pickles, T., & Mróz, L. (2009). The self-management of uncertainty among men undertaking active surveillance for low-risk prostate cancer. Qualitative health research, 19(4), 432-443.

Daubenmier, J. J., Weidner, G., Marlin, R., Crutchfield, L., Dunn-Emke, S., Chi, C., ... & Ornish, D. (2006). Lifestyle and health-related quality of life of men with prostate cancer managed with active surveillance. Urology, 67(1), 125-130.

Would you consider the provision of relaxation and yoga exercises relevant as an intervention to prevent or reduce anxiety, uncertainty and (dis)stress in patients with prostate cancer during active surveillance?

|  |
| --- |

Remarks?

|  |
| --- |

Domain 4: Lifestyle

# Intervention:

Group-oriented relaxation and meditation course. Exercises are offered in 8-10 meetings and participants are invited to do them at home. In the course exercises and meditations are done that enable the participants to orientate themselves and become acquainted with different forms of meditation. In this way they can discover their own way of relaxing and meditating and learn to shape their daily lives.

# Rationale:

A group-oriented course in relaxation and meditation can help care recipients with prostate cancer to find physical and mental relaxation in daily life while actively following a policy. The course offers tools on how to deal with worry and tension. In addition, the course facilitates contact with fellow sufferers between the participants.

Nederlandse vereniging voor psycho-oncologische zorg. (2015). INTERVENTIONS IN DE PSYCHOSOCIALE ONCOLOGISCHE ZORG

Oliffe, J. L., Davison, B. J., Pickles, T., & Mróz, L. (2009). The self-management of uncertainty among men undertaking active surveillance for low-risk prostate cancer. Qualitative health research, 19(4), 432-443.

Daubenmier, J. J., Weidner, G., Marlin, R., Crutchfield, L., Dunn-Emke, S., Chi, C., ... & Ornish, D. (2006). Lifestyle and health-related quality of life of men with prostate cancer managed with active surveillance. Urology, 67(1), 125-130.

Would you consider the use of a group-oriented relaxation and meditation course relevant as an intervention to prevent or reduce anxiety, uncertainty and (dis)stress in patients with prostate cancer during active surveillance?

|  |
| --- |

Remarks?

|  |
| --- |

Domain 4: Lifestyle

# Intervention:

Providing mindfulness instruction and exercises so that care recipients can practice this on a daily basis unsupervised, individually.

# Rationale:

Mindfulness exercises can help patients with prostate cancer to find physical and mental relaxation in their daily life during active surveillance.

Victorson, D., Hankin, V., Burns, J., Weiland, R., Maletich, C., Sufrin, N., ... & Brendler, C. (2017). Feasibility, acceptability and preliminary psychological benefts of mindfulness meditation training in a sample of men diagnosed with prostate cancer on active surveillance: results from a randomized controlled pilot trial. Psycho‐oncology, 26(8), 1155-1163.

Would you consider the provision of mindfulness exercises relevant as an intervention to prevent or reduce anxiety, uncertainty and (dis)stress in patients with prostate cancer during active surveillance?

|  |
| --- |

Remarks?

|  |
| --- |

Domain 4: Lifestyle

# Intervention:

The use of motivational interviewing by (oncology) nurses to promote a healthy lifestyle. Aimed at achieving a healthy BMI, promoting exercise and/or smoking cessation.

# Rationale:

Obesity and smoking are associated with an increased risk of life-threatening prostate cancer and a poorer prognosis. According to the studies by Dickeran (2017) and Brookman-May (2019), the advice of urologists in men with prostate cancer should be to achieve a healthy BMI and to stop smoking. Motivational interviewing can help care recipients to change behavior. A healthy lifestyle contributes to positive coping with prostate cancer and active policy.

Dickerman, B. A., Ahearn, T. U., Giovannucci, E., Stampfer, M. J., Nguyen, P. L., Mucci, L. A., & Wilson, K. M. (2017). Weight change, obesity and risk of prostate cancer progression among men with clinically localized prostate cancer. International journal of cancer, 141(5), 933-944.

Brookman-May, S. D., Campi, R., Henríquez, J. D., Klatte, T., Langenhuijsen, J. F., Brausi, M., ... & Minervini, A. (2019). Latest evidence on the impact of smoking, sports, and sexual activity as modifiable lifestyle risk factors for prostate cancer incidence, recurrence, and progression: a systematic review of the literature by the European Association of Urology Section of Oncological Urology (ESOU). European urology focus, 5(5), 756-787.

Do you think the use of motivational interviewing for a healthy lifestyle is relevant as an intervention to prevent or reduce fear, uncertainty and (dis)stress in care recipients with prostate cancer during active surveillance?

|  |
| --- |

Remarks?

|  |
| --- |
